# Eco-epidemiology of porcine trypanosomosis in Karim Lamido, Nigeria: prevalence, seasonal distribution, tsetse density and infection rates

**DOI:** 10.1186/s13071-016-1732-x

**Published:** 2016-08-12

**Authors:** Solomon Ngutor Karshima, Ikwe Ajogi, Garba Mohammed

**Affiliations:** 1 Department of Veterinary Public Health and Preventive Medicine, University of Jos, PMB 2084 Jos, Nigeria; 2 Department of Veterinary Public Health and Preventive Medicine, Ahmadu Bello University, PMB 1045 Zaria, Nigeria; 3 Department of Veterinary Surgery and Medicine, Ahmadu Bello University, PMB 1045 Zaria, Nigeria

**Keywords:** Eco-epidemiology, Prevalence of porcine trypanosomosis, Seasonal distribution, Tsetse density and infection rates, Species-specific PCR

## Abstract

**Background:**

Animal trypanosomosis is a major economic disease in Nigeria causing considerable morbidity and mortality in livestock. Despite reports in other animals, the disease is under reported in pigs.

**Methods:**

We conducted a community based epidemiological study on African animal trypanosomosis in Karim Lamido area of Taraba State, Nigeria using species-specific PCR on 712 pigs and 706 of the 2822 captured tsetse flies. Data were analysed using Chi-square, odds ratio and multivariate analysis at 95 % confidence interval.

**Results:**

Overall prevalence of porcine trypanosomosis was 16.6 % and ranged between 2.0 and 8.8 % across *Trypanosoma* species. Seasonal distribution of porcine trypanosomosis varied significantly (*χ*^2^ = 16.62, *df* = 3, *P* = 0.0008) ranging between 7.9 and 23.6 % across seasons. Mixed infections involving *T. b. brucei*, *T. congolense* forest and *T. congolense* savannah recorded infection rates ranging between 2.5 and 9.3 %. There were significant variations between the trypanosome infection rates in relation to age (*χ*^2^ = 7.629, *df* = 1, *P* = 0.0057, OR = 1.932, 95 % CI = 1.203–3.100), sex (*χ*^2^ = 10.09, *df* = 1, *P* = 0.0015, OR = 2.085, 95 % CI = 1.315–3.304) and body condition (*χ*^2^ = 22.10, *df* = 2, *P* < 0.0001) of pigs ranging between 10.4 and 30.3 %. Tsetse infection rates were 11.2 % (79/706) for *Glossina palpalis* and 6.8 % (48/706) for *G. tachinoides* yielding an overall infection rate of 18.0 %.

**Conclusion:**

*Trypanosoma* species are prevalent in the study area with similar distribution patterns in both pigs and tsetse flies. Late rainy season, adults, females and pigs with poor body condition recorded higher trypanosome infection rates. Of the three *Trypanosoma* spp. identified, *T. b. brucei* showed predominance.

## Background

Trypanosomoses refer to a group of vector-borne parasitic diseases caused by protozoa of the genus *Trypanosoma. Trypanosoma brucei brucei*¸ *T. congolense*, *T. vivax*, *T. evansi*, *T. simiae* are all infective to animals causing African animal trypanosomosis while *T. brucei gambiense* and *T. brucei rhodesiense* are the only species pathogenic to man in Africa. Transmission is usually cyclical through bites of infected flies of the genus *Glossina*; however, mechanical transmission is possible by other haematophagous flies of the genera *Hamatopota*, *Tabanus* and *Stomoxys.*

Tsetse-transmitted trypanosomoses occur in 38 sub-Saharan African countries with less than 10,000 human cases and one million cattle deaths reported yearly, exposing over 70 million people and 160 million cattle to the risk of infection in the region [1]. The disease is distributed over a wide range of habitats covering about 10 million square kilometres of potential grazing and farming lands in sub-Saharan Africa [2]. It is one of the major factors militating against the development of livestock industry in Nigeria. Substantive evidence shows that, 21 of the 38 countries endemic for trypanosomosis were grouped among the 25 poorest countries of the world indicating the role of the disease in Africa’s struggle against poverty [3].

In Nigeria, tsetse-transmitted trypanosomosis is endemic in over 80 % of the 928,300 km^2^ land-mass and follows the pattern of tsetse distribution covering the area between latitudes 4° and 13°N of the country including the highlands of Jos, Mambilla and Obudu which were earlier considered tsetse- and trypanosomosis-free [4]. The most important *Trypanosoma* species in Nigeria are *Trypanosoma brucei brucei*, *T. congolense*, *T. vivax* and *T. evansi* in animals as well as *T. brucei gambiense* which is infective to humans [5]. Economic losses caused by these parasites as a result of mortality, loss in productivity, costs of treatment and other control programmes were estimated at over 4.2 million USD annually [5].

Despite efforts to eradicate poverty through livestock production, several factors including trypanosomosis are still militating against the livestock sector in Nigeria. Trypanosomosis is well documented in Nigeria in cattle [6–8], small ruminants [9–11] and camels [12, 13]. However, there is paucity of information on porcine trypanosomosis despite the role of this animal species in the economy of the nation. In this study, we determined the prevalence of porcine trypanosomosis, tsetse density and infection rates in Karim Lamido, Nigeria.

## Methods

### Study area

**Fig. 1:**
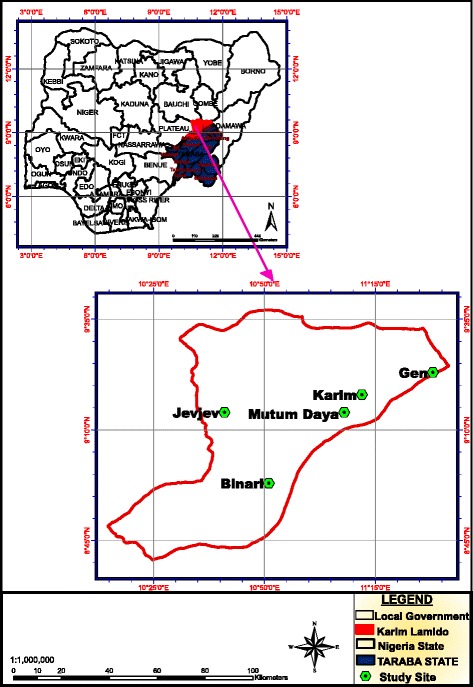
Map of Nigeria showing Taraba State, Karim Lamido and the study sites

This study was carried out in Karim Lamido Local Government Area of Taraba State which is located in North-eastern Nigeria between latitudes 8°33′–10°21′N and longitudes 10°21′–11°24′E (Fig. 1). It covers a land mass of 6,620 km^2^ with a population of 195,844 and a sub-Sudan vegetation. It is bounded to the south by the River Benue and traversed by several tributaries of the same river. It has two distinct seasons namely; rainy which extends from May to October and dry which extends from November to April with an average annual precipitation of 1058 mm and annual average temperature of 28 °C. The major agricultural activities in the region include crop farming, livestock production and fishing. Karim Lamido is a centre for pig trade and pigs are bred under intensive, semi-intensive and extensive management systems.

### Study design

We conducted a community-based cross-sectional study of porcine trypanosomosis between December, 2013 and September, 2014 in five villages (Binari, Gen, Jevjev, Karim and Mutum Daya) located at radii of at least 5 to 10 km apart. For the purpose of our study, we divided the year into four seasons namely; late dry (February - April), early rainy (May - July), late rainy (August - October) and early dry (November - January) and a total of 712 pigs were sampled across this period. We identified and randomly selected 35 piggeries which were considered as clusters from which individual pigs were selected using simple balloting. These clusters were also sub-divided into sub-units (strata) based on age, body condition and sex of pigs. We also sampled 30 % or 50 % of pigs from piggeries with sizes of ≥ 50 or < 50 pigs respectively taking into consideration strata such as age, sex, season and body condition of pigs. Body condition scores were estimated based on the prominence or absence of protrusion of the bones of the ribs, pin-bone and spinous processes of the backbone in to thin (poor), borderline (intermediate) and optimum (good) as earlier described [14].

Tsetse flies were trapped using twelve biconical traps mounted 120 m apart, thrice a week during December, 2013, March, June, and September, 2014. Trypanosome infection rates in both pigs and tsetse flies were estimated at 95 % confidence intervals.

### Blood sampling of pigs and purification of trypanosomes

Five ml of blood was aseptically collected from each pig via venopuncture and transferred immediately into clean labelled sample bottles containing ethylene diamine tetra-acetic acid (EDTA) at 1.5 mg/ml [15, 16]. This was gently shaken until the blood was properly mixed with the anticoagulant.

The trypanosomes isolated from pig blood were separated from the blood using a DEAE 52 column (Whatman, Maidstone, Kent, UK) as described by [17] and stored at 4 °C in phosphate saline glucose (PSG) buffer until needed for DNA extraction.

### Entomological studies

Tsetse flies were trapped by the use of biconical traps as described by [18]. Traps were emptied every 24 h and flies were identified using morphological characteristics as described by [19]. Following identification, one fourth of the total catch which was randomly selected across seasons and locations were stored at -80 °C and later subjected to species specific PCR for the identification of vector stages of trypanosomes.

### Genomic DNA extractions

Trypanosome DNA was extracted from pig blood and tsetse flies using GeneJET genomic DNA extraction kit (Thermo Scientific, Germany). Briefly, 200 μl of purified trypanosomes was lysed by adding 400 μl of lysis solution and 20 μl of proteinase K as recommended by the manufacturer. For extraction of genomic DNA from tsetse flies, the tsetse flies were first homogenized and further subjected to the DNA extraction protocol for trypanosomes as described above. Extracted DNA was stored at -20 °C until needed for PCR.

### Molecular identification of trypanosomes

We conducted eight different PCRs namely *Trypanosoma brucei* (TBR), *T. congolense* forest (TCF), *T. congolense* kilifi (TCK), *T. congolense* savannah (TCS), West African *T. vivax* (WA-TV), *T. evansi* (TeRoTat), *T. gambiense* serum glycoprotein (TgsGP) and Serum resistant antigen (SRA) PCRs. All PCRs were carried out in 25 μl reaction mixture containing 1U Taq DNA Polymerase (Promega, USA), PCR buffer (Promega, USA), 2.5 mM MgCl_2_ (Promega, USA), 200 μM of each of the four dNTPs (Roche, Mannheim, Germany), 1.0 μl of primers in Table 1 and 5.0 μl of sample DNA. Amplifications were carried out using Gene Amp PCR system 9700 (Applied Biosystems, UK). Reaction conditions for the different PCRs were as shown in Table 2 and all amplified products were analyzed by electrophoresis in a 2 % agarose gel and UV illumination after ethidium bromide staining. DNAs extracted from different animal trypanosomes obtained at the National Institute for Trypanosomiasis Research, Jos, Nigeria, and human infective trypanosomes obtained at the Institute of Tropical Medicine, Antwerp, Belgium were used as positive controls while water was used as a negative control.

**Table 1:** Primer names, targets, sequences and amplicon sizes for PCR detection of African trypanosomes

Primer [Reference]	Target species	Target gene	Sequence (5'-3')	Amplicon size (bp)
TBR1 [44]	*Trypanozoon*	177 bp repeat	cgaatgaatattaaacaatgcgcagt	173
TBR2 [44]		Sequence	agaaccatttattagctttgttgc	
TCK1 [45]	*T. c.* kilifi	SDNAm	gtgcccaaatttgaagtgat	294
TCK2 [45]			actcaaaatcgtgcacctcg	
TCF1 [45]	*T. c.* forest	SDNAm	ggacacgccagaaggtactt	350
TCF2 [45]			gttctcgcaccaaatccaac	
TCS1 [45]	*T. c.* savannah	SDNAm	cgagaacgggcactttgcga	316
TCS2 [45]			ggacaaagaaatcccgcaca	
TVWI [46]	*T. vivax*	ILDat 1.2	gtgctccatgtgccacgttg	175
TVW2 [46]			catatggtctgggagcgggt	
TeRoTat 920 F [47]	*T. evansi*	RoTat 1.2	ctgaagaggttggaaatggagaag	151
TeRoTat 1070R [47]		VSG gene	gtttcggtggttctgttgttgtta	
TgsGP-F [48]	*T. brucei*	TgsGP	gctgctgtgttcggagagc	308
TgsGP-R [48]	*gambiense*		gccatcgtgcttgccgctc	
SRA-F [49]	*T. brucei*	SRA	atagtgacaagatgcgtactcaacgc	284
SRA-R [49]	*rhodesiense*		aatgtgttcgagtacttcggtcacgct

**Table 2:** Reaction conditions for different polymerase chain reactions

PCR	PD Temp/time	No. of cycles	DTemp/time	T*m* Temp/time	ETemp/time	FE Temp/time
TBR	94 °C, 3 min	35	94 °C, 30 s	55 °C, 1 min	72 °C, 1 min	72 °C, 5 min
TCF, TCK and TCS	94 °C, 3 min	35	94 °C, 1 min	60 °C, 2 min	72 °C, 1 min	72 °C, 5 min
WA-TV	94 °C, 1 min	35	94 °C, 30 s	55 °C, 1 min	72 °C, 2 min	72 °C, 5 min
TeRoTat	95 °C, 2 min	35	95 °C, 30 s	58 °C, 30 s	72 °C, 1 min	72 °C, 5 min
TgsGP and SRA	95 °C, 15 min	45	95 °C, 1 min	63 °C, 1 min	72 °C, 1 min	72 °C, 5 min

*Abbreviations*: *PD* (Pre-denaturation), *D* (denaturation), *Tm* (annealing temperature), *E* (extension), *FE* (final extension), *Temp* (temperature)

### Data analysis

Data collated were analyzed using Statistical Package for Social Sciences (SPSS 20.0) and Graph-Pad Prism 4.0. Trypanosome infection rates in pigs were calculated by dividing the number of pigs positive for trypanosomes by the total number of pigs examined and expressed as percentages. Tsetse infection rates were calculated by dividing the number of infected tsetse flies by the total number of flies analysed and expressed as percentages. Average tsetse catch per day was determined by summing the daily catch per week and dividing by the number of days traps were mounted for the week while average catch per trap was determined by summing daily catch and dividing by the number of traps. The Chi-square (*χ*^2^) test and odds ratio were employed where appropriate to determine statistical associations between prevalence rates of different variables examined. We also employed multivariate analysis to determine variations in the seasonal distributions of porcine trypanosomosis and tsetse infection rates at 95 % confidence interval and values of *P* < 0.05 were considered significant.

## Results

**Fig. 2:**
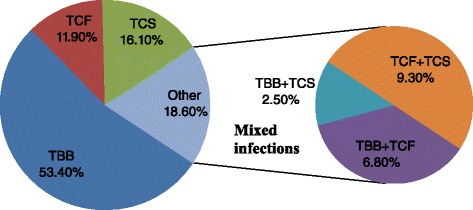
Distribution of *Trypanosoma* species among the 118 positive pigs. *Abbreviations*: TBB, *T. b. brucei*; TCF, *T. congolense *forest; TCS, *T. congolense *savannah; TBB + TCF, *T. b. brucei *and *T. congolense* forest mixed infections; TBB + TCS, *T. b. brucei* and *T. congolense* savannah mixed infections; TCF + TCS, *T. congolense* forest and *T. congolense* savannah mixed infections

We analysed a total of 712 pig blood samples across seasons, 178 each during the early dry (December), late dry (March), early rainy (June) and late rainy (September) seasons. A total of 118 pigs were positive for *Trypanosoma* species yielding an overall trypanosome infection rate of 16.6 %, of which 8.8 % (63/712), 2.0 % (14/712), 2.7 % (19/712) and 3.1 % (22/712) were *T. brucei brucei, T. congolense* forest*, T. congolense* savannah and mixed infections, respectively (Fig. 2; Table 3). Seasonal distribution of swine trypanosomosis varied significantly (*χ*^2^ = 16.62, *df* = 3, *P* = 0.0008) and were 16.3 % (29/178), 7.9 % (14/178), 18.5 % (33/178) and 23.6 % (42/178) for early dry, late dry, early rainy and late rainy seasons, respectively (Table 3). Of the 22 (18.6 %) mixed infections detected, 2.5, 6.8 and 9.3 % were due to *T. b. brucei* and *T. congolense* savannah, *T. b. brucei* and *T. congolense* forest as well as *T.congolense* forest and *T. congolense* savannah (Fig. 2).

**Table 3:** Seasonal distribution of porcine trypanosomosis in Karim Lamido

Season	Number examined	Number positive (%)	*T. b. brucei* (%)	*T. congolense* forest (%)	*T. congolense* savannah (%)	Mixed infections (%)
Early dry	178	29 (16.3)	15 (8.4)	4 (2.3)	6 (3.4)	3 (1.7)
Late dry	178	14 (7.9)	8 (4.5)	1 (0.6)	2 (1.1)	2 (1.1)
Early rainy	178	33 (18.5)	18 (10.1)	6 (3.4)	4 (2.3)	11 (6.2)
Late rainy	178	42 (23.6)	22 (12.4)	3 (1.7)	7 (3.9)	6 (3.4)
Total	712	118 (16.6)	63 (8.8)	14 (2.0)	19 (2.7)	22 (3.1)

Age (*χ*^2^ = 7.629, *df* = 1, *P* = 0.0057, OR = 1.932, 95 % CI = 1.203–3.100) and sex (*χ*^2^ = 10.09, *df* = 1, *P* = 0.0015, OR = 2.085, 95 % CI = 1.315–3.304) based trypanosome infection rates varied significantly and were 17.2 % (93/484), 15.4 % (25/228) 19.9 % (91/458) and 10.6 % (27/254) for adults, piglets, females and males, respectively (Table 4). There was also significant variation (*χ*^2^ = 22.10, *df* = 2, *P* < 0.0001) between the infection rates of 15.1 % (63/417), 10.4 % (18/173) and 30.3 % (37/122) recorded by pigs with good, intermediate and poor body conditions, respectively (Table 5).

**Table 4:** Age and sex based prevalence of porcine trypanosomosis in Karim Lamido

Variable	Number examined (%)	Number positive	Prevalence (%)	*P*-value (*χ* ^2^)	Odds ratio (95 % CI)
Age					
Adults (> 6 months)	484 (67.9)	93	17.2	0.0057	1.932
Piglets (≤ 6 months)	228 (32.1)	25	15.4	(7.629)	(1.203–3.100)
Total	712 (100)	118	16.6		
Sex					
Female	458 (64.3)	91	19.9	< 0.0001	
Male	254 (35.7)	27	10.6	(10.09)	2.085
Total	712 (100)	118	16.6		(1.315–3.304)

**Table 5:** Prevalence of porcine trypanosomosis in relation to body condition of pigs

Body condition	Number examined (%)	Number positive	Prevalence (%)	*P*-value (*χ* ^2^)
Good	417 (58.6)	63	15.1	
Intermediate	173 (24.3)	18	10.4	< 0.0001
Poor	122 (17.1)	37	30.3	(22.10)
Total	712 (100)	118	16.6	

A total of 2822 tsetse flies were trapped within 4 months with overall apparent density of 4.9 tsetse flies per trap per day. Of the tsetse flies caught, 32 flies were found dead during collection from the traps and 28 were teneral flies (Table 6). None of the teneral tsetse flies were included among the 706 flies analysed for trypanosome identification. Apparent densities per seasons were 3.4, 3.9, 5.0 and 7.3 flies/trap/day for early dry, late dry, early rainy and late rainy season, respectively (Table 6). We subjected 706 (25.0 %) of the total tsetse flies caught to species-specific PCR for the presence of vector stages of trypanosomes. We obtained tsetse overall infection rate of 18.0 % (127/706), of which 11.2 % (79/706), 6.8 % (48/706) represented infection rates in *Glossina palpalis* and *Glossina tachinoides,* respectively (Table 7).

**Table 6:** Apparent densities and infection rates of tsetse flies per season

Season	Catch/trap/day	Total catch	No. of dead flies (%)	No. of teneral flies	No. of subjected to PCR	No. of infected (%)	GP (%)	GT (%)
Early dry	3.4	485	12 (2.5)	3 (0.6)	122	10 (8.2)	7 (5.7)	3 (2.5)
Late dry	3.9	568	7 (1.2)	5 (0.9)	142	18 (12.7)	6 (4.2)	12 (8.5)
Early rainy	5.0	713	6 (0.8)	9 (1.3)	178	37 (20.8)	23 (12.9)	14 (7.9)
Late rainy	7.3	1056	7 (0.7)	11 (1.0)	264	62 (23.5)	43 (16.3)	19 (7.2)
Total	4.9	2822	32 (1.1)	28 (1.0)	706	127 (18.0)	79 (11.2)	48 (6.8)

*Abbreviations*: *GP*, *Glossina palpalis*; *GT*, *Glossina tachinoides*

**Table 7:** Tsetse infection rates in relation to species of trypanosome

Tsetse species	No. trapped	No. subjected to PCR (%)	No. infected (%)	TBB (%)	TCF (%)	TCS (%)	MI (%)
*G. palpalis*	1772 (62.8)	451 (63.9)	82 (18.2)	41 (9.1)	23 (5.1)	12 (2.7)	7 (1.6)
*G. tachinoides*	1049 (37.2)	255 (36.1)	45 (17.7)	22 (8.6)	11 (4.3)	6 (2.4)	5 (2.0)
Total	2822 (100)	706 (100.0)	127 (18.0)	63 (8.9)	34 (4.8)	18 (2.6)	12 (1.7)

*Abbreviations*: *TBB,* *Trypanosoma brucei brucei*; *TCF,* *T. congolence* forest; *TCS,* *T. congolense* savannah; *MI,* mixed infections

Of the 127 tsetse flies positive for trypanosomes, 8.9 % (63/706), 4.8 % (34/706), 2.6 % (18/706) and 1.7 % (12/706) were due to *T. b. brucei*, *T. congolense* forest, *T. congolense* savannah and mixed infections, respectively (Table 7). The distribution of trypanosomes in *G. palpalis* were 9.1 % (41/451), 5.1 % (23/451), 2.7 % (12/451) and 1.6 % (7/451) for *T. b. brucei*, *T. congolense* forest, *T. congolense* savannah and mixed infections, respectively while those for *G. tachinoides* were 8.6 % (22/255), 4.3 % (11/255), 2.4 % (6/255) and 2.0 % (5/255), respectively (Table 7).

## Discussion

To the best of our knowledge, this is the first time trypanosomes are characterised in this region using polymerase chain reaction. The findings of the present study are confirmation of the existence of *Trypanosoma* species including *Trypanosoma brucei*, *T. congolense* and *T. vivax* which were earlier reported in this region using conventional parasitological techniques [6, 20]. However, this is the first time a study will identify subspecies and types of trypanosomes like *T. brucei brucei*, *T. congolense* forest and *T. congolense* savannah which the conventional parasitological techniques were unable to identify.

Though our study targeted porcine trypanosomosis, we included primers for the detection of human-infective *T. b. gambiense* and *T. b. rhodesiense* to rule out their presence among all samples that were positive for *T. brucei* (*sensu lato*) using TBR-PCR, especially with recent reports that showed serological [21–23] and molecular [21, 24, 25] evidence of animal reservoirs of human pathogenic trypanosomes. In order to rule out the presence of mechanically transmitted *T. evansi* among the trypanosomes detected in the pigs sampled, we used TeROTat-PCR. Substantive evidence also showed that certain strains of *T. vivax* do not commonly infect pigs [26–28]. Considering the fact that the parasite is reported in and around the study area in ruminants [29–31], their absence in pigs may suggest that the strains present in this region might not be infective to pigs.

We believe that factors including environmental conditions such as humidity and temperature as well as the migration of cattle, which may be another possible source of trypanosomes are associated with the occurrence of tsetse flies and animal trypanosomosis in the region we studied as earlier observed [7, 32]. The overall prevalence of 16.6 % observed in this study was higher than reports from earlier studies [6, 20] that utilized less sensitive diagnostic techniques around the same region. Our finding was, however, lower than the 40.0 and 46.8 % documented by Anosike et al. [33] and Majekodunmi et al. [7], respectively in Jos, Nigeria which was not surprising considering that the work of Anosike et al. [33] was conducted during an outbreak of trypanosomosis. Seasonal distributions of trypanosomes showed significant variations as earlier reported [7]. Rainy season provides ideal environmental conditions for tsetse movements and activity which in turn leads to the transmission of trypanosomosis. This may explain the higher trypanosome infection rates observed in pigs during the rainy season. Other factors which might have contributed to these seasonal variations may be differences in production systems which may include extensive, intensive and semi-intensive as well as environmental conditions including temperature, humidity, vegetation and human activity.

Our finding showed predominance of *T. brucei brucei* over *T. congolense* forest and savannah types in pigs contrary to earlier reports [7, 33] in cattle on the Jos Plateau which revealed predominance of *T. congolense* over *T. b. brucei.* This suggests that different hosts and vectors combine to support trypanosome population within the ecosystem. Although the majority of studies on animal trypanosomosis in Nigeria focused on cattle and small ruminants, *T. brucei* preference for pigs was earlier documented [27, 34]. The epidemiological implications of finding mixed infections in pigs is the risk of tsetse flies transmitting more than one trypanosome species from one infected host to another. This is particularly of concern, considering the varying response of *Trypanosoma* species to different trypanocides. Drug resistance, treatment failure and relapse arising from mixed infections of trypanosomes have also been documented [35].

We sampled 484 (67.9 %) adult pigs against 228 (32.1 %) piglets which did not give equal representation in the sampling because of the unwillingness of pig owners to allow access to the piglets due to their belief that the piglets will be harmed in the process of bleeding. Furthermore, because these pigs were kept for economic purposes, the majority of sampled animals were predominantly females. The higher prevalence observed in females may be due to the stress associated with hormonal imbalances during pregnancy and lactation which usually increases females’ susceptibility to infections.

The majority of the pigs sampled had good body condition; however, pigs with poor body conditions recorded higher prevalence rate which was not surprising since trypanosomosis may also present as chronic and wasting disease in animals and man. *Trypanosoma b. brucei* parasitaemia develops faster than *T. congolense* thus presenting faster effects which we believe is the possible explanation for their predominance among pigs with poor body conditions.

The tsetse fly species reported in this study were earlier reported in the same region [36], as well as other parts of Nigeria [32, 37, 38] and were among the first eleven species of tsetse flies reported in Nigeria [39]. *Glossina palpalis* was predominantly higher than *G. tachinoides* in line with earlier reports [38, 40, 41]. The predominance of *Glossina palpalis* may be associated with factors such as the availability of sources of animal blood meals, temperature, humidity, and other environmental conditions that promote their breeding. The aforementioned factors including the forested nature of the study area may also explain the low tsetse mortality rate observed. In addition, their low fecundity may be a possible explanation for the fewer number of teneral tsetse flies captured.

The study revealed an overall tsetse apparent density of 4.9 flies per trap per day which is slightly lower than the report of Dede et al. [32] who worked around the same periods of the year in Jos, Nigeria and is also within the range of 0.61–8.1 reported elsewhere [41, 42]. The finding of higher tsetse density during the rainy season is in line with the report of Desta et al. [43] in Ethiopia and may be associated to the favourable weather during the season which usually supports tsetse activity thus increasing vector-host contacts and possible transmission of trypanosomes.

This is not the first time that these species of trypanosomes (*T. b. brucei* and *T. congolense*) are reported in this region, suggesting their endemicity and the need to review tsetse control programmes in the region. The distributions of *Trypanosoma* species among domestic pigs and tsetse flies showed a similar pattern which was not surprising because these flies are responsible for the transmission of trypanosomes to the animal species in the region. The majority of trypanosome infections were observed among *Glossina palpalis* contrary to an earlier report [42]. This variation may be due to factors such as differences in feeding frequencies, feeding patterns and vectoral capacity of the tsetse flies. The mixed infections of *Trypanosoma* species observed in tsetse flies may be due to tsetse acquiring the infection from animals carrying the mixed infection in nature or from two different animal sources during feeding.

## Conclusion

*Trypanosoma* species are prevalent in Karim Lamido with similar distribution patterns in both pigs and tsetse flies. Subspecies and types of trypanosomes like *Trypanosoma brucei brucei*, *T. congolense* forest and *T. congolense* savannah are reported for the first time in this region. Late rainy season, adults, females and pigs with poor body condition recorded higher trypanosome infection rates and *T. b. brucei* showed predominance of all the three *Trypanosoma* species identified. *Glossina palpalis* showed predominance over *G. tachinoides* and showed the highest infection rate. It is pertinent to improve on tsetse and trypanosomosis control strategies in the study area so as to curtail economic losses caused by this disease.

## Abbreviations

CI, confidence interval; DEAE, di-ethyl-amino ethanol; df, degree of freedom; DNA, deoxyribonucleic acid; MgCl_2_, magnesium chloride; OR, odds ratio; PSG, phosphate saline glucose; SPSS, statistical package for social sciences, SRA, serum resistance antigen; TBR, *Trypanosoma brucei*; TCF, *Trypanosoma congolense* forest; TCK, *Trypanosoma congolense* kilifi; TCS, *Trypanosoma congolense* savannah; TgsGP, *Trypanosoma gambiense* serum glycoprotein; UV, ultra-violet; WA-TV, West African *Trypanosoma vivax*
